# An Energy Balance Clustering Routing Protocol for Intra-Body Wireless Nanosensor Networks

**DOI:** 10.3390/s19224875

**Published:** 2019-11-08

**Authors:** Juan Xu, Yan Zhang, Jiaolong Jiang, Jiali Kan

**Affiliations:** School of Electronics and Information, Tongji University, Shanghai 201804, China; 1830732@tongji.edu.cn (Y.Z.); 1631515@tongji.edu.cn (J.J.); 1730703@tongji.edu.cn (J.K.)

**Keywords:** intra-body wireless nanosensor networks, terahertz communication, clustering, routing protocol

## Abstract

Wireless NanoSensor Networks (WNSNs) are a new type of network that combines nanotechnology and sensor networks. Because WNSNs have great application prospects in intra-body health monitoring, biomedicine and damage detection, intra-body Wireless NanoSensor Networks (iWNSNs) have become a new research hotspot. An energy balance clustering routing protocol (EBCR) is proposed for the intra-body nanosensor nodes with low computing and processing capabilities, short communication range and limited energy storage. The protocol reduces the communication load of nano-nodes by adopting a new hierarchical clustering method. The nano-nodes in the cluster can transmit data directly to the cluster head nodes by one-hop, and the cluster head nodes can transmit data to the nano control node by multi-hop routing among themselves. Furthermore, there is a tradeoff between distance and channel capacity when choosing the next hop node in order to reduce energy consumption while ensuring successful data packet transmission. The simulation results show that the protocol has great advantages in balancing energy consumption, prolonging network lifetime and ensuring data packet transmission success rate. It can be seen that EBCR protocol can be used as an effective routing scheme for iWNSNs.

## 1. Introduction

Nanotechnology plays an increasingly important role in many fields such as environment, industry, biomedicine and military with its rapid development. Subsequently, the concepts of nano-communication and Wireless NanoSensor Networks (WNSNs) were proposed [1], nano-devices can communicate cooperatively and share the perceived information. Due to the significant advantages in size, biocompatibility and biostability of nanodevices, WNSNs have great application prospects in intra-body health monitoring and medical treatment. Thus, intra-body Wireless NanoSensor Networks (iWNSNs) have also become a new research hotspot.

The development of new materials such as graphene [2] and carbon nanotubes (CNT) [3] that can work at terahertz frequencies (0.1–10 THz) opens new opportunities for applying these nanodevices to the body. This underutilized terahertz spectrum [4,5,6,7] will have a major impact on future medical technologies. First, it can provide a wide bandwidth and the antenna size can be made small. Second, it is less sensitive to propagation effects such as scattering and it is relatively safe for biological tissues [8,9]. Therefore, terahertz wireless communication is an ideal choice for iWNSN physical layer technology.

As one of the key technologies of WNSNs, routing protocols have been studied by scholars in recent years. Currently, researchers mainly study three types of protocols: flooding protocols, proximity routing protocols and energy harvesting-based routing protocols.

The flooding protocol is very simple, which is consistent with the characteristics of limited nano-node energy and computational power [10]. However, flooding protocols can cause broadcast storms and excessive retransmissions, thereby increasing energy consumption.

Proximity routing protocols attempt to improve the performance of flooding protocols by controlling the number of neighboring nodes. Examples of these protocols include CORONA [11], SFR [12] and EEMR [13]. CORONA is a geographic flooding protocol that assumes that nodes in a nanonetwork consist of two types: anchor nodes and user nodes. Anchor nodes have higher communication and processing capabilities than user nodes. User nodes need to localize their location relative to these anchor nodes. The scheme assumes a square fixed network topology in which four anchor nodes are located at the vertices of the square network. A selective flooding routing (SFR) based on terahertz band communication is proposed in [12]. In order to avoid waste of bandwidth resources caused by repeated forwarding of the same data packet, the protocol optimizes the flooding direction. The trend of forwarding packets to the destination node is guaranteed, thereby reducing the number of messages in the network. Xu et al. proposed a protocol called EEMR [13] to solve the problem that the routing protocols such as CORONA and SFR do not consider the terahertz channel attenuation characteristics. However, the EEMR protocol is a single-path routing protocol, which has poor adaptability to network topology changes. For example, it cannot solve the problems that the nano-nodes’ failures due to energy exhaustion or the temporary interruption of the transmission link caused by molecular group disturbance of the channel environment.

Energy-based routing protocols [14,15,16] are specifically designed for self-powered nanonetworks. The main goal of these protocols is to balance energy harvesting and energy consumption, so that the lifetime of the network tends to infinity. In designing such routing protocols, the tradeoff between the complexity and accuracy of the protocol should be considered. For example, Pierobon et al. proposed a routing framework based on energy harvesting [14]. Because of the adoption of the energy harvesting mechanism, the consideration of the tradeoff between energy harvesting and energy consumption, and the establishment of multi-hop routing based on the transmission characteristics of the terahertz channel, the routing protocol can maximize the throughput of the network while ensuring that the network lifetime is infinite. Using the energy harvesting mechanism to make the network lifetime infinite is a major innovation in this protocol. However, the protocol proposed in this paper is too complicated. In the simulation, the maximum number of hops is two, which is not enough for the practical application of the routing in WNSNs. Mohrehkesh and Weigle take into account the current state of energy collected by the communication nodes, and proposed an energy model based on the Markov decision process for data propagation [15]. However, due to the complexity of the proposed model, the authors adopted a lightweight heuristic scheme. Xu et al. proposed a multipath routing protocol EHMR [16] based on energy harvesting. The protocol uses a piezoelectric energy harvesting system to break through the bottleneck of node energy and quantify the contribution of nodes joining paths through the game routing model and establishes multiple feasible paths to improve the stability of the transmission. However, in the route maintenance phase of the EHMR protocol, the forwarding failure is handled by switching the route, and the delay is exchanged for the transmission reliability, which is not applicable to the iWNSN with high delay requirement.

At present, the research on routing protocols is still in infancy, although [17] proposed a cluster-based network topology, the proposed routing proposal did not consider the characteristics of the intra-body nanonetwork firstly, and secondly there was no reasonable cluster head node update mechanism. In addition, the author did not give a routing decision process for nodes between clusters. Therefore, we will study the routing protocol of iWNSNs in this paper that considers the special application environment in the human body.

Due to the small size of the nanosensors, the calculation and processing capabilities of the nano-nodes are limited, and the energy they store is also very limited. However, the nanosensor is very easy to deploy because of its small size. Usually, many nano-nodes are deployed in the target area to ensure that enough data can be collected to complete the monitoring task. Due to the large scale of the network and the small communication range of the nano-nodes, clustering is used to realize data transmission. 

In response to the above problems, we propose an Energy Balance Clustering Routing protocol (EBCR). The protocol adopts a new layered clustering method to reduce the communication load of the nano-nodes, and it also can reduce the number of member nodes in the cluster, and the data packet within the cluster can be transmitted to the cluster head in one hop. In addition, by continuously updating the cluster head node, the node with higher residual energy is selected as the cluster head, which prevents the cluster head node from exhausting energy due to frequent data forwarding, and can also balance the energy consumption in the network. Finally, the cluster head nodes transmit data to the nano control node through multi-hop routing. When selecting the next hop, the distance and the channel capacity is compromised to ensure that the data packet is successfully transmitted while reducing the energy consumption.

The remainder of this paper is organized as follows. In Section 2, the system model is introduced. Next, the clustering routing framework is described in Section 3. In Section 4, we introduce the simulation scenario and define four evaluation indicators: average residual energy, number of dead nodes, data packet transmission success rate and control overhead to evaluate the performance of the new EBCR protocol. Following this, we demonstrate and analyze simulation results for the performance of EBCR protocol in Section 5. Finally, we conclude this paper in Section 6.

## 2. System Model

The computational processing capacity of nanosensors is limited. Many nano-nodes must communicate cooperatively to ensure the normal operation of wireless nanosensor networks. Therefore, iWNSN in our paper is designed as a layered clustering structure. The nano-nodes are deployed in the target area to perceive the data in the environment. The small volume limits its storage and computing capacity, and can only communicate for short distances. Therefore, the nodes in the cluster can transmit data to the cluster head by one hop. Then, the cluster head nodes transfer data to the nano controller (NC) through multi-hop, which has larger volume and stronger calculation processing capability than the nanonode. The NC’s main task is to control nano-nodes by broadcasting simple commands, combine the collected data and ultimately forward it to the nanointerface device.

### 2.1. Network Model

**Figure 1:**
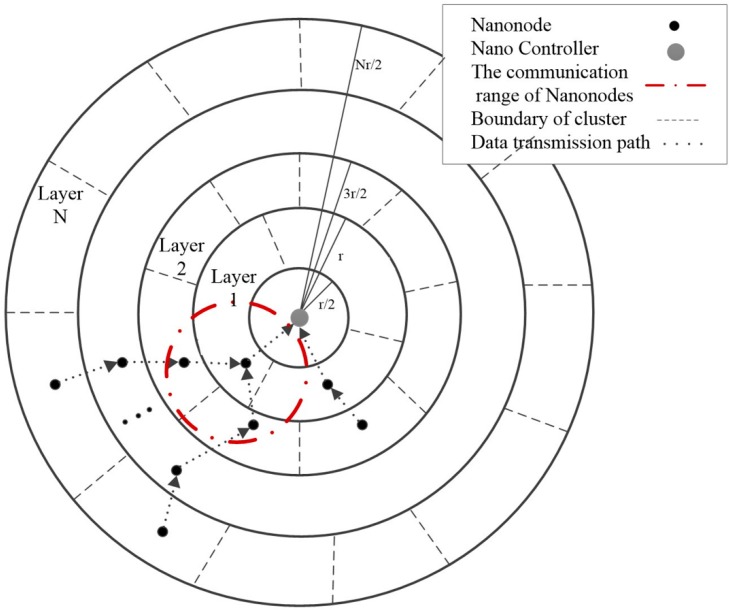
Layered clustering structure of intra-body Wireless NanoSensor (iWNSNs).

In the EBCR protocol, the network topology of iWNSNs can be represented as *G* = (*V*,*E*), where *V =* {*v*_1_, *v*_2_, *…*, *v_n_*} represents a set of all nano-nodes and nano control nodes, and *n* = |*V*| is the number of nodes. The proposed routing framework (shown in Figure 1) is a multi-layer topology based on the distance between nano-nodes and nano control nodes. In order to improve the stability of the network and achieve high energy efficiency, each layer is divided into multiple clusters to avoid repeated clustering. The cluster head node (Cluster Header, CH) of each layer is selected based on the residual energy of the nano-nodes. The cluster head collects data from the cluster member node (CM) and then forwards the summary data to the NC. The clusters of each layer can communicate with adjacent upper or lower clusters.

The network model has the following assumptions:

- The positions of the nano-nodes and the nano control nodes are fixed, and the energy of NC is not limited.
- The terahertz communication of the physical layer of iWNSNs uses TS-OOK modulation, which is like the impulse radio ultra-wideband (IR-UWB) technology, which can accurately locate, and the nano-nodes can obtain the location information of other nodes by broadcasting a simple message.
- In the process of perceiving, processing and transmitting data, the energy consumption of nano-nodes is mainly concentrated in data transmission. The energy consumed by the nano-nodes due to the perceiving and processing data is ignored in the energy consumption model.

### 2.2. Intra-Body Terahertz Channel Model

The path loss of the terahertz channel consists of three parts: the loss experienced by the terahertz wave propagating in the medium, the loss caused by the molecular absorption and the scattering loss during signal propagation [18] Therefore, the path loss can be expressed as:

(1) $$PL\left(f,d\right)=PL_{spr}\left(f,d\right)\times PL_{abs}\left(f,d\right)\times PL_{sca}\left(f,d\right)$$

where PL(f,d) is the total path loss, $PL_{spr}\left(f,d\right)$,$\;PL_{abs}\left(f,d\right)$ and $PL_{sca}\left(f,d\right)$ represent the propagation loss, molecular absorption loss and scattering loss respectively.

Propagation loss represents the transmission loss of electromagnetic waves in a physical medium and can be modeled by a path loss formula Friis, which is defined as:

(2) $$PL_{spr}\left(f,d\right)={\left(\frac{4\pi dn_{m}}{\lambda_{0}}\right)}^{2}$$

where *d* is the propagation distance, $\lambda_{0}$ is the free space wavelength and $n_{m}$ represents the refractive index of human tissue. At the frequency of 1 THz ($\lambda_{0}=300\;\mu m$), the propagation loss is quite high, severely limiting the transmission range of the nanonode. Therefore, in the human body scenario, the communication distance between the nanonode and NC is drastically reduced.

Molecular absorption loss refers to the attenuation caused by molecular absorption. Usually, when electromagnetic waves propagate through the material, a portion of the electromagnetic energy radiated by the nano-antenna is converted to the internal kinetic energy of the molecule. This process depends on the operating frequency and the biological tissue considered. For example, the absorption loss caused by propagation in the skin or blood is different. Thus, the molecular absorption of each material can be characterized by an absorption loss factor α(f), which varies with frequency. Therefore, given α(f), the molecular absorption loss can be obtained by the following formula:

(3) $$PL_{abs}\left(f,d\right)=e^{\alpha \left(f\right)d}$$

Human tissue is composed of different types of substances. The deflection of the beam caused by this microscopic inhomogeneity will cause scattering of particles and affect the propagation of electromagnetic waves. The path loss due to scattering can be expressed as:

(4) $$PL_{sca}\left(f,d\right)=e^{\mu d}$$

where *d* is the propagation distance, and μ is the scattering loss factor. It can be seen from [19] that the scattering loss factor is too small compared to the molecular absorption loss factor, which is almost negligible. Therefore, the path loss can be expressed as:

(5) $$PL\left(f,d\right)={\left(\frac{4\pi dn_{m}}{\lambda_{0}}\right)}^{2}e^{\alpha \left(f\right)d}$$

The phenomenon of molecular absorption from the medium not only affects the characteristic of the channel in terms of attenuation, but also introduces noise. This is the main noise in the terahertz band. The power spectral density of molecular absorption noise can be expressed as:

(6) $$N\left(f,d\right)=K_{B}T_{0}\left(1-e^{-\alpha \left(f\right)d}\right)$$

where *K_B_* represents the Boltzmann constant and *T*_0_ represents the reference temperature.

According to Shannon theory, the channel capacity can be calculated by the following formula:

(7) $$C=Blog_{2}\left(1+\frac{S}{N}\right)$$

where *B* represents the total bandwidth of the system and *S/N* represents the signal-to-noise ratio.

However, the terahertz channel is frequency selective and the noise is non-white noise, so the entire bandwidth is divided into many narrow sub-bands. These sub-bands are very narrow, so the sub-band channels are non-selective and the noise therein is white noise. Therefore, the capacity of each sub-band within the available bandwidth will be calculated to obtain the total channel capacity, so the channel capacity can be written as [20]:

(8) $$C=\underset{i}{\sum }\Delta f\;log_{2}\left(1+\frac{S\left(f_{i}\right)}{PL\left(f_{i},d\right)N\left(f_{i},d\right)}\right)$$

where *S* is the power spectral density of the transmitted signal and the *i*th sub-band is centered around frequency $f_{i}$, having width Δf.

### 2.3. Energy Consumption Model

The energy consumption mainly focuses on the energy consumed by the nano-nodes to receive and transmit data. The energy consumption of the nano-nodes in receiving and transmitting data in the TS-OOK based terahertz channel can be expressed as [19]:

(9) $$E_{tx}\left(N_{bit}\right)=\omega N_{bit}E_{ptx}$$

(10) $$E_{rx}\left(N_{bit}\right)=N_{bit}E_{prx}=\frac{1}{10}N_{bit}E_{ptx}$$

where $E_{tx}\left(N_{bit}\right)$ and $E_{rx}\left(N_{bit}\right)$ represent the energy consumed by the nano-nodes to transmit and receive the $N_{bit}$ bits data packet respectively and $E_{ptx}$ and $E_{ptx}$ represent the energy consumed to transmit and receive the single pulse respectively. The value of ω is related to the coding weight, indicating the probability that the symbol "1" appears in the $N_{bit}$ bits data. ω is usually set to 0.5 in order to make the probability of the symbol "1" and the symbol "0" the same. In a terahertz communication system based on TS-OOK modulation, the received energy consumption is usually set to 1/10 of the transmitted energy consumption [19]. The energy consumption of transmitting unit-bit data can be expressed as:

(11) $$E_{bit}=\omega E_{ptx}+E_{prx}$$

where $E_{bit}$ represents the energy consumed by a node in the transmission link *p* to transmit and receive unit bit data. If the node is a source node which means the information the node senses needs to transmit to the control node, it is considered $E_{prx}=0$, if the node is a destination node, it is considered $E_{ptx}=0$. The energy consumption for transmitting and receiving a single pulse $E_{ptx}$ and $E_{prx}$ are set according to the terahertz channel parameter setting based on TS-OOK modulation in [21]. When the nano-nodes’ communication range is 2 mm, the values of the energy consumed by transmitting and receiving a single pulse in equation (11) are fixed at 1 and 0.1 pJ.

## 3. Clustering Routing Framework

### 3.1. Network Layering

Layering the network helps nano-nodes find the layers they belong to. Assuming that the monitoring area is circular, the entire network is divided into several virtual layers as the NC is set to be center. The width of each layer is *r/2*, the radius of the *l*_th_ layer is (l+1)r/2, where *l* is the number of layers, and *r* is the communication range of each nanonode. This way of dividing layers ensures that the transmission range of each nanonode can cover its adjacent layers. When a broadcast message is received from the NC, all nano-nodes calculate their distance from the NC, compare their distance to the layer radius, and register themselves to the appropriate layer. The algorithm of Nanonode Distribution over Layers (NDL) is given in Algorithm 1.

**Algorithm1:** NDL Algorithm**Notations:** $v_{k}$**=** No. of deployed nanosensors $d_{k}$ = Distance of nanosensor $v_{k}$ with respect to NC $L(v_{k})$ = Layer number to which node $v_{k}$ is assigned r
**=** Transmission range of each nano-node **Data: **r,
$v_{k}$ **Result: **$L(v_{k})$ NC broadcasts “Hello packets” to all $v_{k}$; **foreach**
$v_{k}$
**do**  
$L(v_{k})$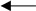 floor($2\frac{d_{k}}{r}$);  
$v_{k}$ registers itself into layer $L(v_{k})$; **end**

The number of clusters of the *l*_th_ layer is

(12) $$\frac{\pi \left[{\left(\frac{l+1}{2}\right)}^{2}r^{2}-{\left(\frac{lr}{2}\right)}^{2}\right]}{\pi {\left(\frac{r}{2}\right)}^{2}}=2l+1$$

### 3.2. Clustering and Cluster Head Updating

After all nano-nodes are assigned to their respective layers, the initial cluster head node is selected based on their remaining energy. In this election process, firstly, the candidate nodes compete for the ability to become the cluster head CH by multicasting their weights to neighbor nodes of the same layer, and the nano-nodes ignore the weights sent by their neighboring layer. In this process, the cluster head node reduces its transmit power to half of the original transmit power to ensure that the diameter of the formed cluster is less than r, thereby ensuring that the node can reach any other nodes in the cluster in one hop. The weight is represented by $\zeta_{k}$. Its calculation is as follows:

(13) $$\zeta_{k}=\frac{E_{res\_k}}{E_{max}}$$

where $E_{res\_k}$ is the residual energy of nanonode *v_k_*, and $E_{max}$ is the maximum energy.

If a given nanonode does not find another nanonode with a larger $\zeta_{k}$, it declares itself to be the CH. After the CH is selected, clustering begins. The CH multicasts short-range messages (messages that declare itself as the cluster head) to all unclustered nano-nodes within the same layer. Furthermore, each non-member nanonode compares multiple short-range messages it receives, sends a join request to the CH corresponding to the strongest RSSI (Received Signal Strength Indication) short-range message it receives and registers itself as a cluster member CM. This process continues until clusters are formed and all nodes have been assigned to their respective CHs. In order to reduce the clustering overhead, the entire network is only clustered once. After each round of data transmission, only the CHs are updated. The cluster formation algorithm (Nano Cluster Formation, NCF) is shown in Algorithm 2.

**Algorithm 2** NCF Algorithm**Notations:** $v_{k}\;$= No. of deployed nanosensors $\zeta_{k}\;$= normalized residual energy of sensor $v_{k}$ $k_{max}\left(\zeta \right)\;$= Nanosensor with maximum ζ *GN* = General Node **Data: **
$L(v_{k}),\;E_{res\_k},\;E_{max}$ **Result: **Clusters **foreach**
$v_{k}$
**do**  Calculate $\zeta_{k}$ with equation (13);  Multicast $\zeta_{k}$ within associated $L(v_{k})$; **  if**
$k_{max}\left(\zeta \right)$ then     Status
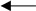
*CH*;  **else**     Status
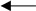
*GH*;  **end** **end** Elected *CH* multicasts short range advert message to *GH* within associated $L(v_{k})$; **foreach**
*GH*
**do**  Check received *RSSI* from all *CHs* within range;  Send join request to *CH* as *CM* for which *RSSI* is maximum and get registered; **end**

In order to prevent the cluster head node from dying due to frequent data forwarding, the cluster head node needs to be updated after each round of data transmission. An adaptive scoring function is used to select and update the cluster head in this paper. In the function, the distance from the node to the cluster head node and the remaining energy of each node are considered. The scoring function is a linear combination of residual energy and node position, and the node with the largest score becomes the cluster head. The scoring function is:

(14) $$S_{k}=w\left(m\right)\frac{E_{res\_k}}{E_{max}}+\left[1-w\left(m\right)\times \frac{d_{\sum^{\;}}-d\left(v_{k},h\left(v_{k}\right)\right)}{d_{\sum^{\;}}}\right]$$

where $S_{k}$ is the score of node $v_{k}$, $E_{res\_k}$ represents the residual energy of node $v_{k}$ and $E_{max}$ represents the maximum energy of node, assuming the initial energy of node is the maximum energy. $h\left(v_{k}\right)$ represents the cluster head node of node $\;v_{k}$, $d_{\sum^{\;}}$ indicates the sum of the distances from all nodes in the cluster to the cluster head node and $d\left(v_{k},h\left(v_{k}\right)\right)$ is the distance from the node $v_{k}$ to the cluster head node, w(m) is the weight coefficient.

The cluster head selection formula is:

(15) $$h^{\prime }=argmax\left(S_{k}\right)$$

where, $h^{\prime }$ is the updated cluster head node.

Considering that the energy of the nodes in the network is decreasing over time, at this time, the chance that the node closer to the original cluster head becomes a cluster head should be increased. Therefore, the weight in (14) is an adaptive weight. As the dead node increases, the weight for the remaining energy is reduced, and the weight for the node position is increased. The mathematical expression is as follows:

(16) $$w\left(m\right)=1-\frac{1}{e^{-\kappa m}}$$

where κ is an adjustable parameter and m is the number of dead nodes in the cluster.

It can be seen from (14), the cluster head update rule expressed is that, the node with greater residual energy and closer the original cluster head node has greater probability to become cluster head. After the candidate node becomes the cluster head, it needs to broadcast the information that becomes the cluster head immediately. With the location information of the cluster head, the remaining nodes can start data transmission.

### 3.3. Communication Mechanism

**Figure 2:**
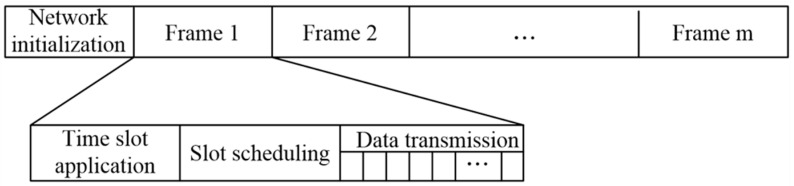
Frame structure.

The medium access layer uses a Carrier Sense Multiple Access with Collision Avoidance (CSMA/CA) and a Time Division Multiple Access (TDMA) hybrid MAC access mechanism. In a conventional wireless network, a node detects whether a channel is busy by means of carrier sensing. In the communication system based on the TS-OOK mechanism, the nodes in the network cannot perform carrier sensing because of the short duration of the pulse. In view of the above problems, the nano-nodes in this paper use the RTS/CTS (Request To Send/Clear To Send) mechanism in CSMA/CA (Carrier Sense Multiple Access/Collision Detection) to examine channel usage and reserve time slots. Since the time slot application packet is small, the probability of collision is relatively low, so the performance of this mechanism is better. In the data transmission phase, the control node NC allocates a total time for each cluster of a specific layer based on the amount of data to be transmitted in the cluster, the time includes the data transmission time of the nodes in the cluster and the time required for the cluster head node to forward the data to the next hop cluster head of the adjacent layer. This method can prevent TDMA-based intra-cluster transmissions from colliding with other clusters from neighboring layers. Based on this hybrid access method, the frame structure can be designed as shown in Figure 2.

**Figure 3:**
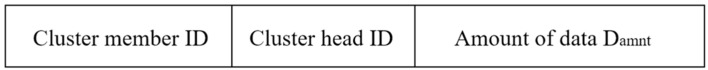
RTS (Request To Send) packet format of cluster member node (CM).

**Figure 4:**
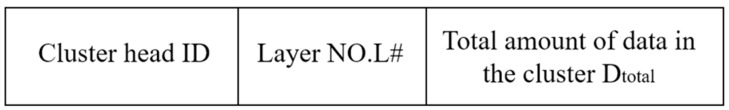
RTS packet format of cluster head node (CH).

In the slot application phase, all cluster member nodes send the slot request packet RTS to the cluster head node, and the format of the RTS packet of the cluster member node is as shown in Figure 3. The $D_{amnt}$ of member node without the data transmission requirement is 0, and the node that needs to transmit data called Active Data Transmission Node (ADTN). If the cluster head node receives the request, it replies a CTS message. Furthermore, if the member node does not receive the CTS after waiting for a certain time, it resends the data request packet. After receiving the data request packet from all member nodes, the cluster head node calculates the total amount of the data that need to be transmitted in the cluster and then randomly selects a neighbor cluster head node from the next layer to forward the RTS packet to the nano control node. The structure of the RTS request packet sent by the cluster head node to the NC is as shown in Figure 4.

After receiving the RTS packets from all cluster head nodes, the NC estimates the total propagation delay required to complete the transmission and specifies the packet transmission order of the ADTN. Transmission time scheduling is divided into the following three phases:

Phase 1: The NC allocates variable length transmission slots for each layer. The length of the time slot depends on the transmission parameters (the total amount of data to be transmitted per layer and the total time required for data transmission). The order of the time slots is from the outermost layer to the innermost layer.

Phase 2: The Control Node NC then allocates the total time for each cluster of a particular layer based on the amount of data that needs to be transmitted within the cluster, which prevents TDMA-based intra-cluster transmissions from colliding with other clusters from the same layer.

Phase 3: Based on the time slots allocated for each cluster, each ADTN node and cluster head node within the cluster will be able to allocate new sub-time slots based on the mechanism shown in Algorithm 3. The specific time slot allocation algorithm (Time Slot Allocation, TSA) is shown in Algorithm 3.

**Algorithm 3** TSA Algorithm **Notations:** LL = List of order of layers p = 1,2,⋯n $T_{p}^{L}$ = Time slot for layer L $T_{p,c}^{L}$ = Time slot for cluster c of layer L $T_{p,c,v}^{L}$ = Time slot for Active Data Transmission Node (ADTN) ν of cluster c of Layer L $T_{p,c,CH}^{L}$ = Time slot for CH of cluster c **Data:** Transmission request message **Result:** Variable length time slots for all ADTNs and all CHs NC relays message to CMs requesting transmission; ADTNs send RTS message to CH; CHs send RTS message to NC; Calculate total time T for a complete cycle; Create the order list LL in the order of outer layer to inner layer; **for each**
LL
**do**   Calculate layer time $T_{p}^{L}$;   **for each**
$T_{p}^{L}$ do     Calculate each cluster time $T_{p,c}^{L}$;     **for each**
$T_{p,c}^{L}$
**do**      Calculate ADTN time $T_{p,c,v}^{L}$;      Calculate CH time $T_{p,c,CH}^{L}$;      Allocate time slots accordingly;      **end**    **end** **end**

**Figure 5:**
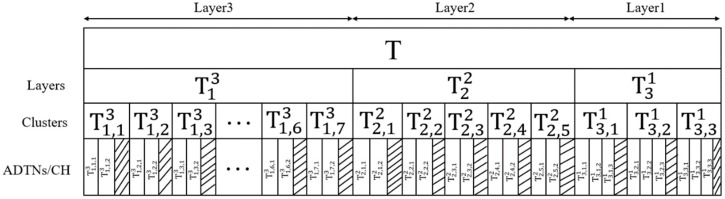
Example of slot scheduling for a three-layer network.

Figure 5 illustrates a time scheduling process for a three-layer network that requires T time to complete the transmission cycle. The time slots are arranged in the order from the outer layer to the inner layer. NC divides the time slot $T_{1}^{3}$, $T_{2}^{2}$ and $T_{3}^{1}$ to Layer 3, Layer 2 and Layer 1 respectively. Layer 1 has three clusters, Layer 2 has five clusters and Layer 3 has seven clusters. Each cluster of Layer 1 has four active nodes ADTN for data transmission, and Layer 2 and Layer 3 for each cluster head have two active nodes. Since the cluster heads between layers need to transmit data, it is necessary to optimize the slot scheduling. For example, the third layer ($T_{1}^{3}$) divides one time slot for each of the seven clusters, and the corresponding time slot numbers are $T_{1,1}^{3},T_{1,2}^{3},T_{1,3}^{3},T_{1,4}^{3},T_{1,5}^{3},T_{1,6}^{3},T_{1,7}^{3}$ respectively. Considering that the inter-layer cluster head needs communication, three sub-time slots $T_{1,1,1}^{3},T_{1,1,2}^{3},T_{1,1,CH}^{3}$ are divided. The first two time slots are used for data transmission of the active node ADTN, but $T_{1,1,CH}^{3}$, the shaded part in the Figure 5 is used for data transmission between the cluster heads. This process is repeated for all clusters and layers. This slot scheduling scheme makes optimal use of the large bandwidth provided by the THz band and avoids transmission collisions at the CH and NC.

In the initial stage, all nano-nodes are in data acquisition mode except for the cluster head node CH. After the time slot allocation is completed, the control node NC sends a wake-up code to activate the outermost layer, thereby activating all ADTNs of the layer for transmission. After receiving all ADTN data, the cluster head node fuses all data and sends it to the next hop node in the time slot allocated to it.

### 3.4. Inter-Cluster Routing

**Algorithm 4:** DTP (Data Transmission Process) Algorithm**Data:**LL **Result:**A Cycle of data transmission **foreach**LL**do**  NC sends wake-up preamble  **while** intra-cluster period **do**   ADTNs send data to respective CH using $T_{p,c,v}^{L}$ time slots:   CH aggregates data;  **end**  **while** inter—cluster—inter—layer period **do**   **if**
$L=1\&\&CH_{c}^{L}$ receives multiple links **then**    $CH_{c}^{L}$ performs fusion;    $CH_{c}^{L}$ transmits fused data to NC;   **else**    $CH_{c}^{L}$ transmit data to lower layer $CH_{c}^{L-1}$ selected    by the next hop selection algorithm;   **end**   New CH is elected using round robin fashions;   New CH multicasts update to other CMs;   Previous CH transfers its information list to new CH;   All ADTNs and previous CH go to harvesting data   except the new CH until another wake-up preamble reaches; **end**

A major problem in iWNSNs is the high probability of link breakdown due to low transmission power and high transmission loss (e.g., path loss). This problem can be successfully solved using collaborative communication between cluster head nodes. When the cluster head choosing next hop to establish a routing, it is always desirable that the next hop node is closer to the nano control node, but this also means that the transmission distance between the cluster head node and the next cluster head node increases. Considering the transmission characteristics of the THz channel, the path loss becomes larger with the increase of the transmission distance and the channel quality deteriorates. Therefore, the link cost function is established as the selection basis of the next hop node, and the candidate path is evaluated. The distance and channel capacity are taken as important factors in the calculation, so that the transmission distance and the channel capacity are compromised. Specifically, the link cost of the candidate path between the cluster head node $h_{i}$ and any of its candidate next hop nodes $h_{j}$ can be calculated by:

(17) $$c\left(h_{i},h_{j}\right)=\lambda \tilde{f}\left(\frac{1}{C_{s}\left(h_{i},h_{j}\right)}\right)+\left(1-\lambda \right)\tilde{f}\left(d\left(h_{j},NC\right)\right)$$

where $d\left(h_{j},NC\right)$ represents the distance between the candidate node $h_{j}$ and the nano control node NC. $C_{s}\left(h_{i},h_{j}\right)$ is the terahertz channel capacity of the candidate path $e\left(h_{i},h_{j}\right)$ between the cluster head node $h_{i}$ and the candidate node $h_{j}$, which can be obtained by the Formula (8). $\tilde{f}()$ represents the result after normalization for each formula, λ represents the cost factor, and satisfies λ∈(0,1). Smaller cost value indicates that the candidate path is better, and the candidate node is more likely to be selected as the next hop.

**Figure 6:**
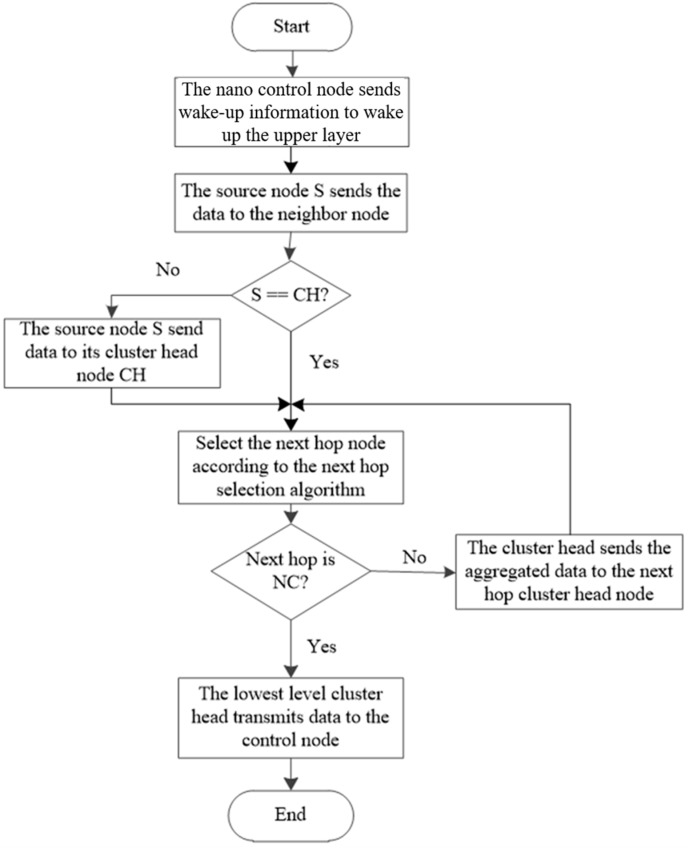
EBCR routing process.

The DTP between clusters and between layers is shown in Algorithm 4. The CH fuses the data and selects the appropriate adjacent lower layer CH according to the link cost function and forwards the data. After forwarding the data, the CH uses the cluster head update algorithm to initiate a new CH election. The new CH then multicasts its new state to other CMs. The previous CH provides its information list to the new CH and enters the data collection mode. All nodes except the new CH start collecting data and suspending the data transfer mode until another wake-up message arrives. The EBCR algorithm process is shown in Figure 6.

## 4. Simulation Scenario and Evaluation Index

This paper uses NS-3 to simulate the performance of the designed routing protocol.

In order to verify the correctness and superiority of the EBCR protocol, we analyze the EBCR protocol, the classical clustering protocol Low Energy Adaptive Clustering Hierarchy (LEACH), MH-LEACH protocol which makes further development in LEACH to select the cluster-heads and cluster formation [22] and Selective Flooding Routing (SFR). LEACH [23] is a classic clustering algorithm in the traditional wireless sensor network. The protocol randomly selects the cluster heads in a round-robin manner, so it can distribute the energy load of the entire network evenly to each sensor node, thereby achieving the purpose of reducing network energy consumption and improving the overall lifetime of the network. Based on the LEACH protocol, MH-LEACH [24] establishes multi-hop communication between sensor nodes in a network with the main goal of saving energy. In MH-LEACH protocol, the cluster heads send the aggregated data to the base station through multi-hop forwarding. In this process, the routing is mainly based on RSSI. SFR [12] is an optimization of the flooding routing, which optimizes the flooding direction and ensures the trend of data packets forwarding to destination nodes, thereby reducing the number of messages in the network. Like the EBCR, the SFR protocol is a multi-hop routing protocol. Therefore, by comparing the EBCR with the SFR protocol, the performance of the network is simulated as the network load increases (the data packet generation interval becomes smaller).

The simulation scene is a circular two-dimensional plane, and the nano-nodes are randomly distributed in the area. First, investigate the energy changes of the network as the simulation time advances. Then the performance variation of the iWNSNs is investigated when the distance between the nanonode and the control node changes.

### 4.1. Simulation Scenario

**Figure 7:**
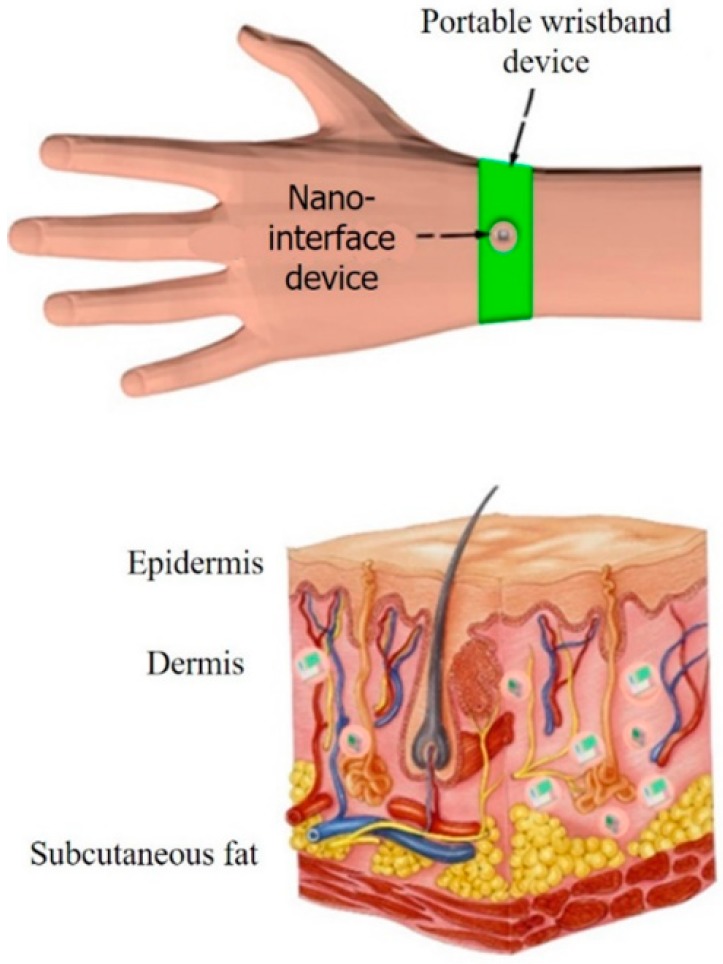
Model of nano-nodes in the skin.

In this paper, when modeling a wireless nanosensor network, it is assumed that nanosensor nodes are placed in the hands to construct iWNSNs. The system architecture is shown in Figure 7. 

The nanointerface device is placed on the back of the hand, and the nano-nodes are implanted into the skin to monitor certain skin indicators. The biological composition of the back of the hand can be modeled as a layered structure, the upper layer consisting of skin, divided into dermis and epidermis. It is demonstrated in [25] that these two human tissues have very similar electrical properties, so in this paper, both are treated as a single tissue. The middle layer represents subcutaneous fat, which is thinner on the back of the hand. This thinness makes the back of the hand ideal for building a body area network because the distance between the outside and the vein is quite short. As described in [26], nanodevices must be encapsulated in biocompatible capsules or artificial cells for implantation into the human body.

### 4.2. Evaluation Index

In the simulation experiment, the network lifetime, average residual energy, data packet transmission success rate, control overhead and effective throughput are selected to analyze the routing protocol. The specific definitions are as follows.

#### 4.2.1. Network lifetime

There are many different definitions of network lifetime. This paper simulates the change of the number of dead nodes in the network with the simulation time, and focuses on the time of the first dead node in the network and the time of the last dead node. Death time (DT) is defined as:

(18) $$DT=\min\left\{t|E_{k}\left(t\right)\leq \eta \right\},\;v_{k}\in V$$

where *DT* represents the death time of the node in the iWNSNs. $E_{k}\left(t\right)$ indicates the energy value of the node $v_{k}$ at the time *t*. η is the threshold value of the remaining energy. When the node energy level is below the threshold, the node can be considered dead.

#### 4.2.2. Average Residual Energy

The average residual energy refers to the average remaining energy of the nodes in the network, which is calculated as follows:

(19) $$\bar{E_{res}}=\frac{\sum_{k}^{n}E_{res\_k}}{n\cdot E_{max}}$$

where $E_{res\_k}$ represents the remaining energy of the node $v_{k}$ at the current moment, *n* represents the number of nodes in the network and $E_{max}$ represents the maximum value of the energy of the node, which takes the initial energy of the nano-node in the simulation.

#### 4.2.3. Data Packet Transmission Success Rate

The data packet transmission success rate can reflect the working efficiency of the routing protocol, which represents the ratio of the total number of successfully received data packets of the destination node to the total number of data packets in the network. The calculation formula is:

(20) $$R_{suc}=\frac{F_{r}}{F_{t}}$$

where $R_{suc}$ is the data packet transmission success rate, $F_{t}$ is the number of data packets sent by the nano nodes in the network and $F_{r}$ is the number of data packets received by the nano control node.

#### 4.2.4. The Proportion of Control Overhead

The proportion of control overhead refers to the proportion of packets used for nano-node control information to total data packets in the network. It can be written as:

(21) $$\eta_{control}=\frac{N_{control}}{N_{control}+N_{service}}$$

where $N_{control}$ is the data packets for control information, $N_{service}$ is the data packets for service information and the total data packets are the sum of $N_{control}$ and $N_{service}$. The larger the value, the greater the proportion of control overhead.

#### 4.2.5. Average End-to-End Delay

There is a processing delay, queuing delay, propagation delay and transmission delay in the transmission of packets. Average end-to-end delay $\bar{T}$ is defined as mean of the time a packet takes from being generated to received successfully at the control node, it can be written as:

(22) $$\bar{T}=T_{pr}+\frac{\sum_{i=1}^{N_{rec}}\left[T_{qi}+\frac{d_{i}}{v}+\frac{L_{data}}{C_{s}\left(d_{i}\right)}\right]}{N_{rec}}$$

where $T_{pr}$ is the processing delay; $T_{qi}$ is the queuing delay; $d_{i}$ is the distance between the node transmitting the data packet and the control node; $L_{data}$ is the size of the data packet sent by the sensing node, in this paper $L_{data}$ of all nodes is the same; v is the propagation speed of the signal in the medium; $C_{s}\left(d_{i}\right)$ is the channel capacity corresponding to the node $v_{i}$; $N_{rec}$ is the number of packets successfully received at the control node.

#### 4.2.6. Effective Throughput

The effective throughput is defined as the number of bits of the data packet successfully received by the destination node per unit time, which can be expressed as:

(23) $$Th_{avg}=\frac{l_{N}N_{bit}R_{suc}}{\Delta T}$$

where $Th_{avg}$ is the network average throughput, $N_{bit}$ is the number of bits in a single packet, $l_{N}$ is the number of data packets during ΔT and $R_{suc}$ is the data packet transmission success rate.

### 4.3. Simulation Parameters

**Figure 8:**
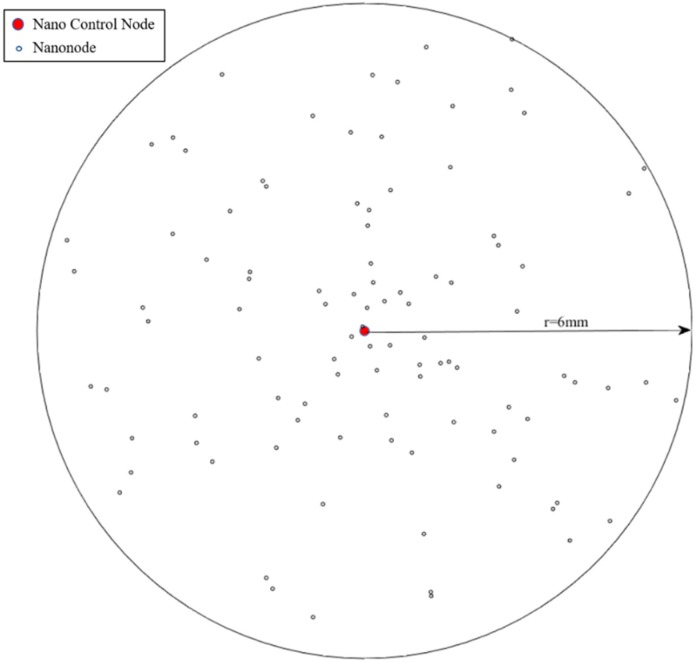
Simulation Scenario.

The simulation scenario in this paper considers the nano-nodes deployed in the skin dermis of the hand to construct a nanonetwork. According to [27], the molecular absorption factor of the skin is 110, the refractive index is 1.73, and the system parameter κ in Equation (16) is set to 0.5, the residual energy threshold η in Equation (18) is set to 1.4 × 10^−13^J [21], which represents the energy consumption required to receive unit bit data when the transmission distance is 0.002 m. The simulation scenario is shown as Figure 8. Nano-nodes are randomly distributed in the area. The remaining specific simulation parameters are set as Table 1 is shown.

Please notice that in Figure 10, we change the number of nano-nodes in the network lifetime simulation, but in other simulation we use 100 nano-nodes to simulate. In the simulation that considers the data packet generation interval as an independent variable, we change the interval in the range of 1 to 10 s, in other simulation, we use a fixed data packet generation interval 0.1 s to simulate.

## 5. Simulation Results and Analysis

### 5.1. Network lifetime

**Figure 9:**
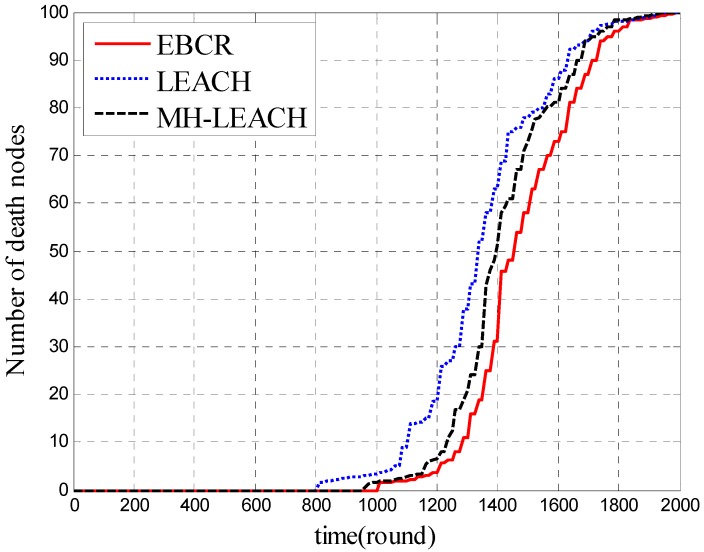
Comparison of the number of dead nodes.

**Figure 10:**
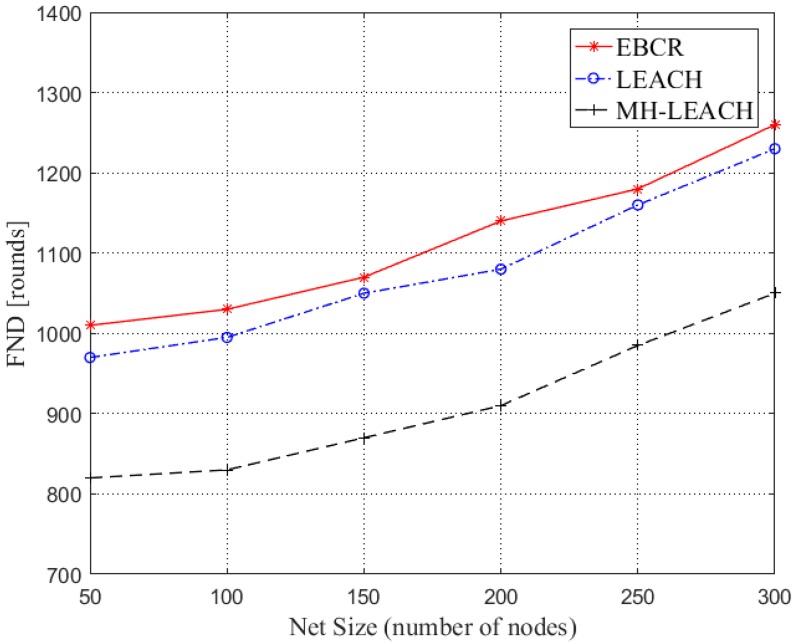
Comparison of the time of first node’s death.

In Figure 9, the network lifetime is analyzed by simulating the number of dead nodes in the network. The simulation time is represented by rounds (each round is 120 s). In Figure 10, the time when the first node is dead is compared. In the simulation, the time refers to the number of iteration round. FND is the abbreviation for First Node’s Death. We choose LEACH and MH-LEACH to compare rather than SFR in this part because SFR has an energy void and there is no need to compare its network lifetime to EBCR. According to Figure 9 and Figure 10, we can see that the EBCR protocol prolongs the death time of the first node in the network and improves the stable transmission time of the data in the network. This is because EBCR preferentially selects the cluster with higher residual energy when selecting the cluster head node while LEACH and MH-LEACH randomly select the cluster head, causing the cluster head node to die prematurely. In addition, both EBCR and MH-LEACH adopt multi-hop forwarding, which can well balance the energy consumption in the network and effectively prevent the node from exhausting energy due to massive data transmission. In general, the EBCR protocol can well balance network energy consumption and extend network lifetime.

### 5.2. Average Residual Energy

**Figure 11:**
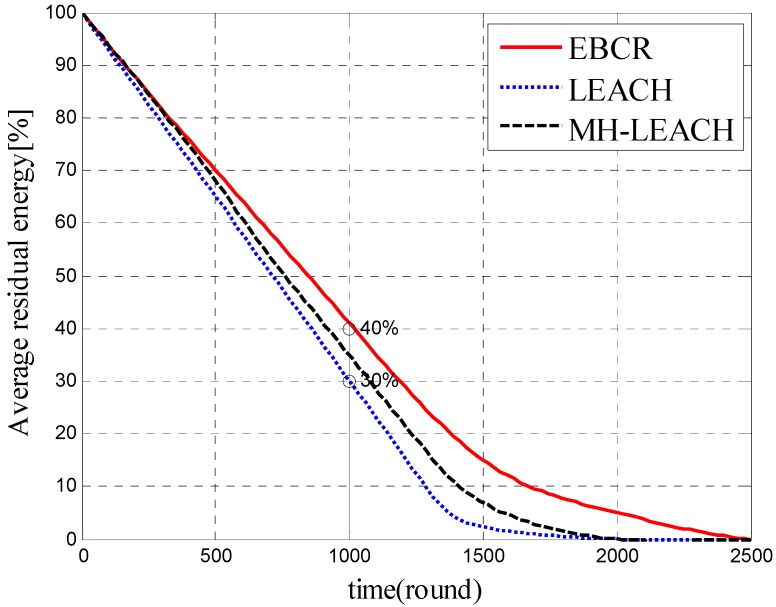
Comparison of average remaining energy.

Figure 11 shows the average residual energy of nodes in the network. As same to the comparation of the network lifetime, we choose LEACH and MH-LEACH to compare rather than SFR in this part because SFR has an energy void and there is no need to compare its average residual energy to EBCR. The percentage of residual energy at each time for the three algorithms can be compared from Figure 11. At the same time, the average residual energy of the EBCR is always higher than that of LEACH and MH-LEACH. This shows that the EBCR protocol is more efficient in saving network energy and improving node energy utilization. 

### 5.3. Data Packet Transmission Success Rate

**Figure 12:**
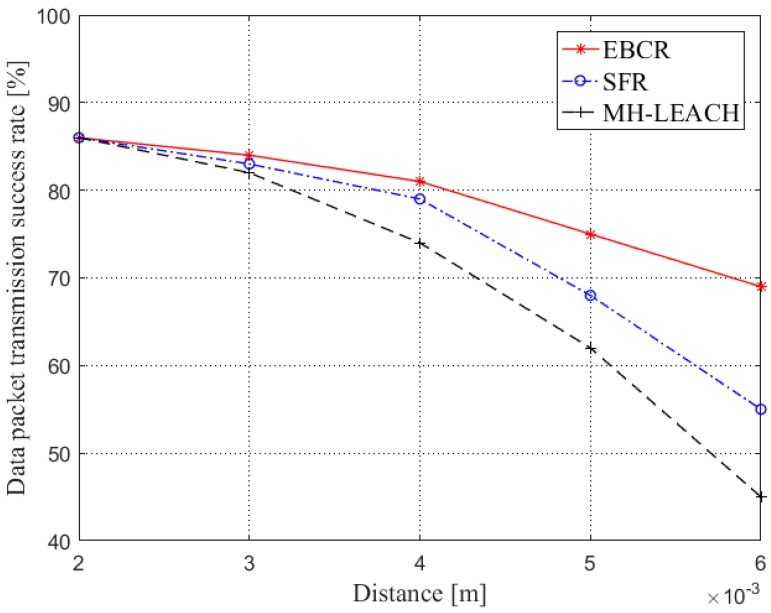
Comparison of the data packet transmission success rate (distance).

Figure 12 shows the comparison of the success rate of data packet transmission in the aspect of the distance. With the increase of the distance between nodes, the transmission success rate of three protocols is decreasing. This is because there is the possibility of packet loss during the frequent forwarding of data. However, the transmission success rate of EBCR and SFR is always kept at a higher level. When the communication distance is greater than 4 mm, the data packet transmission success rate of MH-LEACH decreases, this is because when the communication range increases, the cluster head close to the control node needs to frequently participate in data forwarding. Furthermore, the cluster head update of MH-LEACH is random. When some nodes with less energy are selected as cluster heads and participate in data forwarding, data packets are lost due to exhausted energy, which also reflects the advantages of EBCR’s cluster head update mechanism. 

**Figure 13:**
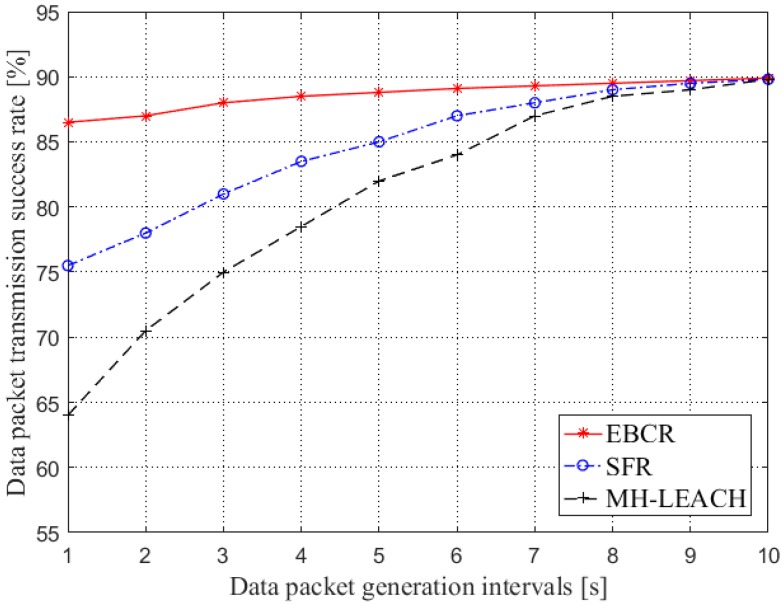
Comparison of the data packet transmission success rate (generation intervals).

Figure 13 shows the comparison of the success rate of data packet transmission in the aspect of data packet generation interval. As can be seen from the figure, the packet transmission success rate of the EBCR protocol is always higher than that of the other two protocols. This is because the protocol ensures the path from the source node to the destination node through hierarchical clustering and cluster head update. The correct forwarding of data packets improves the reliability of the network. In SFR, due to the flooding mechanism, a reliable path can always be found between the source node and the destination node to ensure the success rate of data packet transmission. However, because of this, the frequent participation of nodes in data forwarding also causes the energy consumption of nodes in the network. When many nodes die, it will affect the success rate of data packet transmission. For the MH-LEACH protocol, as the network load increases, the cluster head nodes close to the control node die due to frequent participation in forwarding, resulting in a sharp drop in the success of data packet transmission.

### 5.4. Proportion of Control Overhead

**Figure 14:**
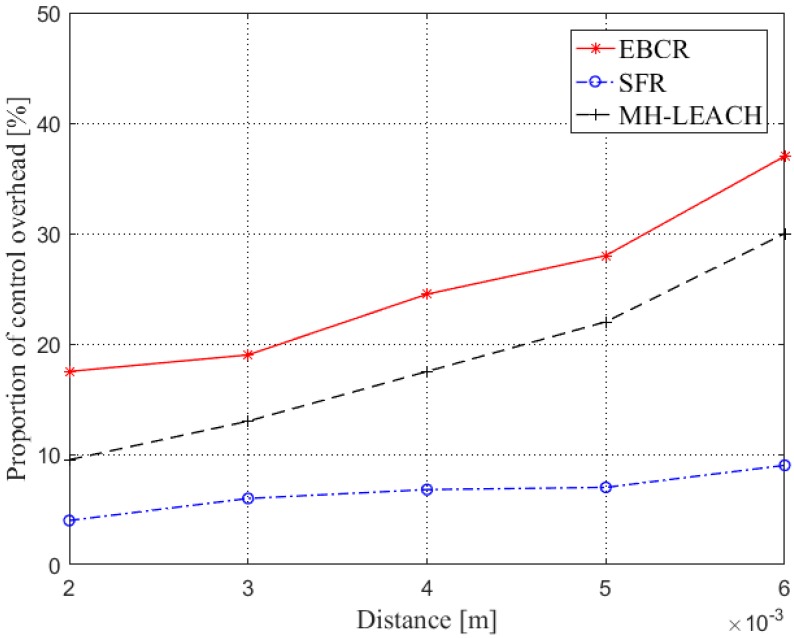
Comparison of proportion of control overhead.

It can be seen from Figure 14 that the proportion of control overhead of the EBCR and MH-LEACH is always higher than that of SFR. Obviously, the control overhead of SFR is lower than that of clustering protocols. In the multi-hop forwarding process of the MH-LEACH protocol, a route needs to be established, which causes a certain control overhead. When the distance between the source node and the control node is small, the routing process of the MH-LEACH protocol is relatively simple, and the control overhead is small. As the distance increases, the process of establishing the route by the MH-LEACH protocol becomes complicated, and the control overhead also increases. Furthermore, because the EBCR protocol stratifies the network before clustering, the control overhead is slightly larger than MH-LEACH. However, it is also through this hierarchical clustering method that the communication load and energy consumption of the nano nodes are minimized while ensuring the successful transmission of data packets.

### 5.5. Average End-to-End Delay

**Figure 15:**
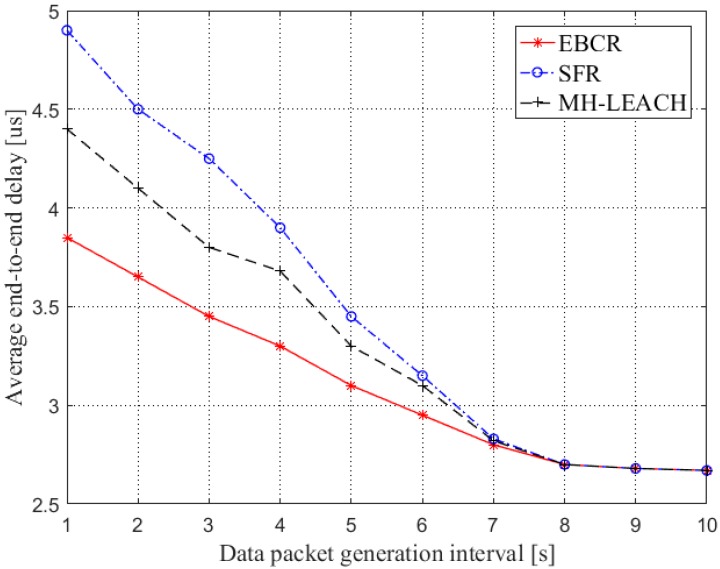
Comparison of average end-to-end delay.

Figure 15 compares the end-to-end delays of the three protocols. In general, the average end-to-end delay is reduced as the packet generation interval increases. This is because when the packet generation interval becomes larger, the number of packets arriving in the node buffer per unit time becomes smaller, and the time at which the packet waits for transmission in the buffer area is shortened, thereby reducing the end-to-end delay. Moreover, when the packet generation interval is large (≥7 s), the average end-to-end delay of the three protocols is relatively close. In addition, the end-to-end delay of the route established by the EBCR protocol is smaller than the other two routes.

### 5.6. Effective Throughput

**Figure 16:**
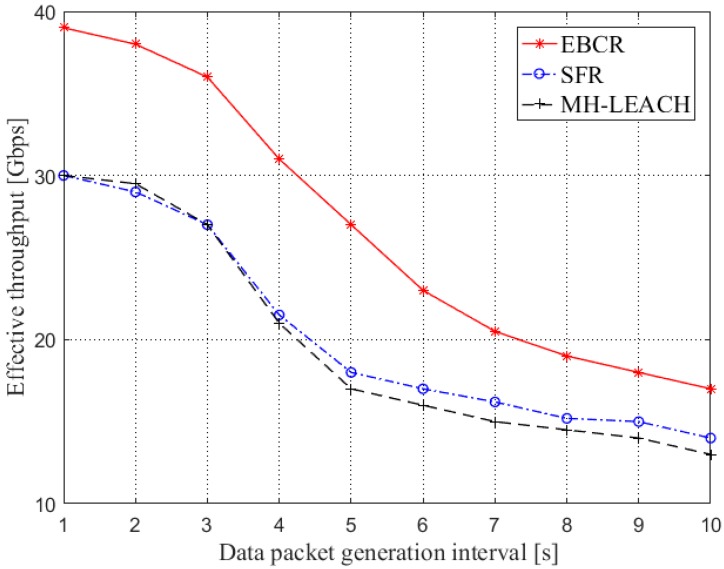
Comparison of effective throughput.

Figure 16 compares the effective throughput of the three protocols. As the interval of data packet generation increases, the effective throughput of the three protocols also decreases. This is because as the interval of data packet generation increases, the number of data bits flowing into the network per unit time decreases. The size of the effective throughput is related to the transmission success rate and the transmission rate of the selected link. According to the previous analysis, the EBCR protocol has a good data packet transmission success rate and a low delay. It can be seen that the EBCR protocol enables the iWNSNs to exhibit a very good network performance.

## 6. Conclusions

In this paper, an energy balance clustering routing protocol (EBCR) is proposed for the intra-body nanosensor nodes with low computational and processing capabilities, short communication range, and limited energy storage. This protocol reduces the energy load of the nano-nodes by adopting a new layered clustering method, reduces the number of member nodes in the cluster, reduces the data transmission distance of the nodes in the cluster and enables the nano-nodes to transmit data to the cluster head node in a hop within the cluster. In addition, by continuously updating the cluster head node, the node with higher residual energy is selected as the cluster head, which prevents the cluster head node from exhausting energy due to frequent data forwarding, and can also balance the energy consumption in the network. Finally, the cluster head nodes transmit data to the nano control node through multi-hop routing. By establishing a link cost function, the next hop is selected to compromise between distance and channel capacity, ensuring successful data packet transmission and reducing energy consumption. In the simulation results, by comparing EBCR with LEACH, MH-LEACH and SFR, it proves that the EBCR has a great advantage in terms of equalizing energy consumption, prolonging network lifetime, ensuring data packet transmission success rate and ensuring a larger effective throughput. Therefore, EBCR protocol can be used as an effective routing scheme for iWNSNs.

## Figures and Tables

**Table 1:** Simulation parameter setting.

Simulation parameter (unit)	Value
Radius of simulation scenario (mm)	6
Number of nano-nodes	100
Number of nano control nodes	1
Physical layer pulse width (fs)	100
Physical layer pulse interval (ps)	10
Data packet size (Byte)	128
Data packet generation interval (s)	0.1, [1:1:10]
Nanonode communication range (mm)	2
Simulation time per round (s)	120
Number of iteration rounds	2500
Distance between source node and control node (mm)	2,3,4,5,6
Nanonode initial energy (μJ)	4
